# Pharmacokinetic interactions of psychotropic medications in patients with schizophrenia suffering from atypical mycobacterial infections

**DOI:** 10.1192/j.eurpsy.2021.1725

**Published:** 2021-08-13

**Authors:** M. Betriu Sabaté, A. González-Rodríguez, A. Guàrdia, E. Pérez-Macho, A. Alvarez Pedrero, S. Acebillo, J.A. Monreal, D. Palao Vidal, J. Labad

**Affiliations:** 1 Mental Health, Parc Tauli University Hospital, Sabadell, Spain; 2 Mental Health, Parc Taulí University Hospital. Autonomous University of Barcelona (UAB). I3PT, Sabadell, Spain; 3 Emergency Service, Hospital Universitario Ramón y Cajal, Madrid, Spain; 4 Mental Health., Hospital Universitari Mútua de Terrassa. CIBERSAM, Terrassa, Spain; 5 Department Of Mental Health, Parc Taulí University Hospital. Autonomous University of Barcelona (UAB). I3PT. CIBERSAM, Sabadell, Spain; 6 Mental Health, Hospital of Mataró. Consorci Sanitari del Maresme. CIBERSAM., Mataró, Spain

**Keywords:** schizophrénia, Mycobacterium, infection, therapeutic drug monitoring

## Abstract

**Introduction:**

Mycobacterium kansasii is a nontuberculous mycobacterium that causes infection associated with past or current tuberculosis disease. Clinical syndromes and radiological findings are mostly indistinguishable from that of Mycobacterium tuberculosis, thus requiring microbiological confirmation.

**Objectives:**

We report a case of a 44-year-old man diagnosed with schizophrenia and Mycobacterium kansasii infection.

**Methods:**

Case report and non-systematic narrative review from PubMed.

**Results:**

Case report: Patient with schizophrenia who was admitted at the inpatient unit presenting psychotic exacerbation with high levels of excitement. Risperidone 6 mg/day and valproate 500 mg/day were initiated. He was also diagnosed with a M. kansasii lung infection, with radiological findings of past tuberculosis disease. Before the microbiological confirmation, it was necessary to start rifampicin, requiring an increase in doses of both psychotropic drugs. Review: (1)Comorbidity of mycobacterial infections and schizophrenia. Several studies have shown that people with severe mental illness have higher rates of tuberculosis compared with the general population. Although the relationship between tuberculosis and M. Kansasii infection is known, few literature is available with regard to the association of M. Kansasii and schizophrenia. (2)Interactions between antipsychotics and mood stabilizers with rifampicin. Rifampicin is mainly metabolized by CYP3A4 and transported by P-glycoprotein. Add-on with rifampicin have been reported to reduce clozapine and olanzapine plasma levels (despite both are metabolized by CYP1A2), reduce haloperidol and risperidone levels (possible role of P-glycoprotein in this interaction), as well as for valproate.

**Conclusions:**

Treatment of comorbid infections in people with schizophrenia remains a challenge. Antibiotics used to treat mycobacterial infections can modify the pharmacokinetic of psychotropic drugs.

**Disclosure:**

No significant relationships.

